# The Perceived Support From Light and Color Before and After an Evidence-Based Design Intervention in an Emergency Department Environment: A Quasi-Experimental Study

**DOI:** 10.1177/19375867221150215

**Published:** 2023-03-02

**Authors:** Jeanette Lindahl, Hans Thulesius, Helle Wijk, David Edvardsson, Carina Elmqvist

**Affiliations:** 1 Centre of Interprofessional Collaboration within Emergency care (CICE), Department of Health Caring Sciences, Linnaeus University, Växjö, Sweden; 2 Department of Research and Development, Region Kronoberg, Växjö, Sweden; 3 Department of Clinical Sciences, Lund University, Malmö, Sweden; 4 Department of Medicine and Optometry, Faculty of Health and Life Sciences, Linnaeus University, Växjö, Sweden; 5 Institute of Health and Care Sciences, Sahlgrenska Academy at Gothenburg University, Sweden; 6 Department of Quality Assurance and Patient Safety, Sahlgrenska University Hospital, Gothenburg, Sweden; 7 Department of Architecture and Civil Engineering, Chalmers University of Technolog, Gothenburg, Sweden; 8 School of Nursing and Midwifery, La Trobe University, Melbourne, Victoria, Australia; 9 Department of Nursing, Umeå University, Sweden

**Keywords:** color, emergency department, evidence-based design, family members, light, multidisciplinary research, patients, physical care environment, self-report questionnaire

## Abstract

**Aim:**

To evaluate patients’ and family members’ perceived support from light and color before, compared with after an evidence-based design (EBD) intervention at an emergency department (ED) using a validated instrument—the Light and Color Questionnaire (LCQ).

**Background:**

EDs offer acute care day and night. Thus, a supportive physical environment where light and color is crucial for how the milieu is experienced is vital. Research is limited on how care settings are perceived as supportive by users.

**Methods:**

Quasi-experimental evaluation of the refurbishing and remodeling of an ED by an expert group of nurse managers, nursing staff, nursing researchers and architects in south Sweden. LCQ includes dimensions “maximizing awareness and orientation,” “maximizing safety and security,” “supporting functional abilities,” “providing privacy,” “opportunities for personal control” (not for LCQ-Color), and “regulation and quality of stimulation.” LCQ was analyzed and compared in 400 surveys from 100 patients and 100 family members before the intervention and 100 patients and 100 family members after the intervention.

**Results:**

The LCQ total score significantly improved after the intervention for both patients and family members. Four of the six dimensions of LCQ Light subscale scores were significantly higher for family members, and three of the six dimensions were significantly higher for patients after the intervention. The LCQ Color subscale score showed significant improvements for all five dimensions for both patients and family members after the intervention.

**Conclusion:**

This study showed improved perceived support from light and color in the physical environment for patients and family members after an EBD intervention at an emergency department using a validated instrument—the Light and Color Questionnaire.

## Background

An emergency department (ED) is a unique place with guaranteed access to acute care day and night (Ajeigbe et al., 2013); therefore, this complex milieu should be accommodating for all users. This highlights the need to create a supportive physical environment where all feel safe and secure, and where unnecessary worry and stress are avoided (Salonen et al., 2013). Overcrowded EDs are often characterized by long wait times, which exacerbate feelings of lack of control, vulnerability, fear, and anxiety (Elmqvist & Frank, 2015; Sonis et al., 2018). The self-image of patient and family member often change when arriving at the ED, where a previously independent, well-informed, and healthy individual can suddenly find themselves being in midst of uncertainty, illness/injury, and in an unfamiliar and unwanted patient/family member role. The perceived self-images of patients and family members often change when arriving at the ED: a previously independent, well-informed, and healthy individual becomes an ignorant, uncertain, ill, or injured patient (Dahlen et al., 2012; Möller et al., 2010).

Light and color in the physical environment have been linked to psychological, physiological, and social reactions affecting the five senses, with a unique impact on each individual, where light and color together have been described as necessary in order to perceive and understand the world (Klarén, 2017; Mahnke, 1996). Natural light and daylight enable an orientation to both the day and the seasons, and they have been described as fundamental to people’s health and well-being. Furthermore, people have described to prefer natural daylight or light that is perceived as natural to artificial light since daylight is linked to time and changes throughout the day (Haans, 2014; Iyendo et al., 2016). Additionally, patients exposed to natural daylight feel less stressed, use less pain medication, and experience better sleep. Softening the physical environment indoors with natural daylight through windows can contribute to a healing environment. Artificial light, however, can cause visual fatigue and headaches. Furthermore, natural light shining through windows facilitates movement and wayfinding, contributes to comfort, satisfaction, well-being, and a sense of independence as well as affecting patient outcomes, such as patient satisfaction and safety (Iyendo et al., 2016).

***Light and color in the physical environment have been linked to psychological, physiological, and social reactions affecting the five senses, with a unique impact on each individual, where light and color together have been described as necessary in order to perceive and understand the world***

Therefore, physical environments should be designed to allow the greatest possible access to daylight (Ulrich, 2012) and to cycle lighting that parallels day and night (Engwall et al., 2015). However, it is not the strength of the light but how humans experience it that determines how the environment is perceived and whether it affects health positively or negatively according to previous studies (Ulrich, 2012;Ulrich et al., 2008; Ulrich et al., 2010).

Human eyes can only see color when light is present. Color is crucial for localization, movement, and wayfinding and is key to recognizing places and contrasts to clarify orientation points (Wijk, 2001, 2006). Moreover, natural colors and materials such as wood can promote well-being and lower stress levels (Ohta et al., 2008). Therefore, muted earth tones with characteristics of earth (brown, beige, and blue), stone (gray), and plants (green) have been described as desirable for use in care environments since the ability to distinguish colors does not change significantly with increasing age (Kellert & Calabrese, 2015). Color coding/contrast is also useful in care settings both to attract and divert attention (Billger & Fridell Anter, 2017).

There is a growing body of evidence suggesting that the physical environment has a significant impact on patients and family members in care situations (Iyendo et al., 2016) and is a resource to utilize for support (van der Zwart, 2021). Notably, this focus on the physical environment as a support (Nightingale, 1859/2003) has developed into theories of supportive and evidence-based design (EBD; Ulrich, 2012; van der Zwart, 2021). Applied EBD promotes healing environments and the integration of data from various research disciplines based on the best available knowledge about health environments and architecture (Ekman et al., 2021; Ulrich, 2012). EBD focuses on the architecture of health environments to promote healing environments that support users, such as patients and family members (Stichler & Hamilton, 2008;Ulrich et al., 2008; Ulrich et al., 2010).

Today, there are several studies of physical environment (light and color) in different care contexts. However, related to daylight versus artificial lightning, wayfinding or/and other factors are related to well-being, satisfaction, sleep, anxiety, pain, length of stay, comfort, and so on (Bayramzadeh et al., 2021; Brambilla et al., 2019; Eijkelenboom & Bluyssen, 2022; Iyendo et al., 2016; McCunn et al., 2021; Schreuder et al., 2016). Furthermore, for several of these studies, were the participants volunteers, that is, not actual patients, studies with real patients and family members are preferable (Jamshidi et al., 2020). Moreover, there is a lack of research evaluating perceived support in physical environments from light and color in terms of awareness, orientation, safety, security, functional abilities, privacy, and opportunities for personal control. The goal of this study was to fill this knowledge gap.

## Aim of the Study

To evaluate patients’ and family members’ perceived support from light and color before, compared with after an EBD intervention at an ED using a validated instrument—the Light and Color Questionnaire (LCQ).

## Materials and Methods

**Figure 1.**
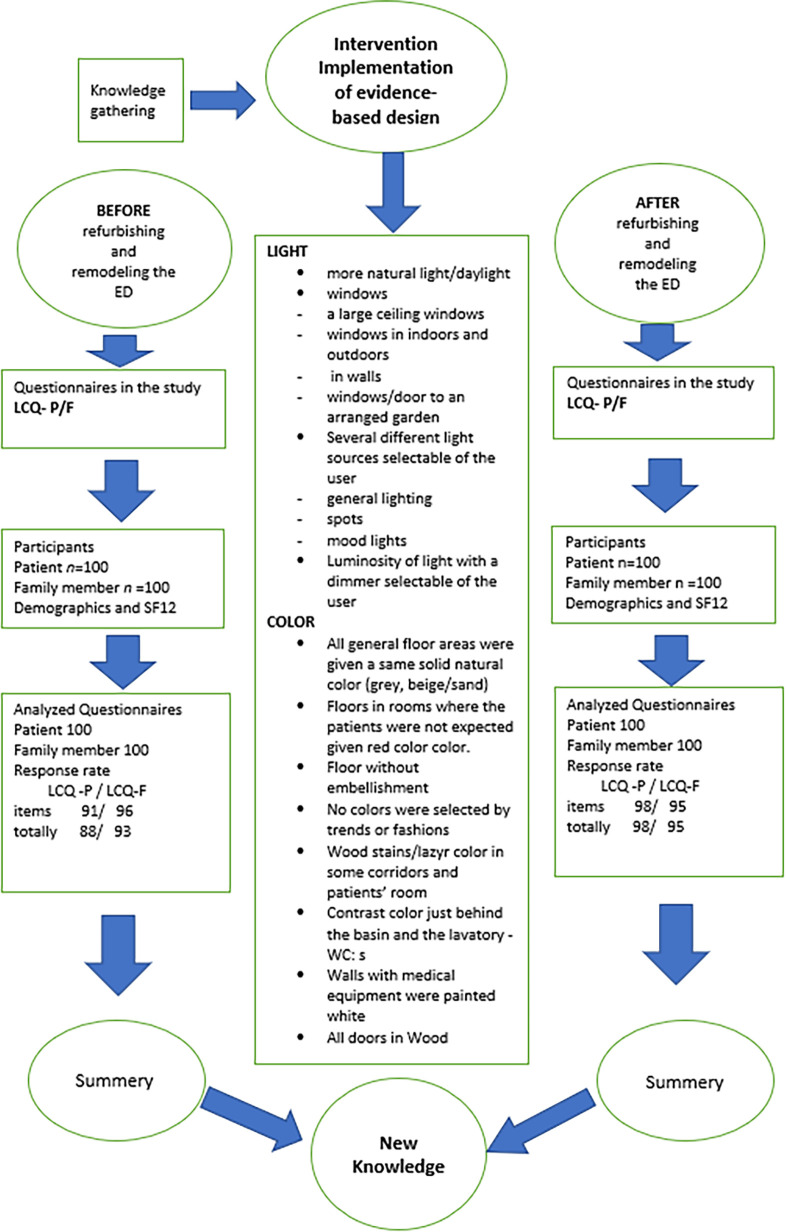
The flow of the participants, SF12, intervention and response rate of Light and Color Questionnaire.

This study employed a quasi-experimental design with preintervention data collected 1 year before the refurbishment and remodeling of an ED and postintervention data collected 1 year after the refurbishment and remodeling of the ED. This design provided the possibility of comparing and evaluating the EBD from light and color intervention outcomes (Figure 1).

### Setting

This study took place at a hospital in south Sweden offering emergency care on a 24/7 basis for 125,000 people. The ED was divided into the following sections: internal medicine, surgery, orthopedics, infectious disease, gynecology, and ear-nose-and-throat diseases. During a typical 24-hr period, the ED treated 80 patients with a 50/50 gender distribution and 90% being 18 years and older.

A triage system with color coding—red-orange-yellow-green-blue (red and yellow = unstable, immediate care, yellow, green and blue = stable)—was used to sort patients (Wireklint et al., 2018). About 20% of the ED patients were triaged red and orange, and 30% of them arrived by ambulance. More than one third of patients had internal medical conditions, one-quarter orthopedic or surgical conditions, and the remainder infectious disease, gynecological, or ear-nose-and-throat diseases. The median total care time per patient was 165 min in 2010 and 156 min in 2012.

Before refurbishment and remodeling, the ED had 13 patient rooms (single rooms), three open patient spaces (for monitoring), one acute room, one working station without daylight, and one waiting room outside the ED. After refurbishment and remodeling, the ED had 21 patient rooms (single rooms), six open patient spaces (for monitoring), two acute rooms, one open working station with a large skylight window, and two waiting rooms—one outside the ED and one internal waiting room.

### Participants

The study included patients and family members who visited the ED between 8:00 a.m. to 8:00 p.m. during the data collection periods (before-and-after studies were carried out in February 2010 and 2012, respectively). Patients and family members were included if they were 18 years or older, could master written Swedish, had enough visual acuity, and had physical and mental ability to personally answer the survey. Exclusion criteria were patients arriving by ambulance or being triaged as red (urgent, highest acuity level) or orange (physician assessment within 30 min). Their condition was thus considered to prevent them from having time and ability to respond to the survey.

#### EBD intervention

The refurbishment and remodeling from EBD intervention was designed based on the teamwork of a multidisciplinary expert group including two assistant nurses, one registered nurse, and three nurse managers from the ED, as well as a research team including the first and last authors, a senior lecturer in nursing, and architects. Data from seminars, study visits, fact-finding visits, regional policies and guidelines, and scientific literature conducted according to the principles and guidelines of EBD were analyzed in the design work process. The EBD intervention focused on altering the lighting and color during the refurbishment and remodeling of the ED.

The EBD intervention goals for lighting were to provide access to increased daylight as well as varied artificial lighting adapted to the users’ needs. The ED was remodeled to have more windows on indoor walls and in all doors to treatment and investigation rooms, which were also furnished with blinds. A large skylight window was placed in the middle of the ED above a central workstation. A large window with a glass door was placed at the rear of the ED, providing access to a courtyard and garden. By using several different light sources, such as general lighting, spot lighting, and mood lighting as well as brightness dimmer in the patient rooms and waiting rooms, the ability of patients and other users to control and choose a light source was increased.

***The EBD intervention goals for lighting were to provide access to increased daylight as well as varied artificial lighting adapted to the users’ needs.***

The EBD intervention goals for color were to use colors to make it easier for users to find their way and gain information, thereby increasing safety and security. All common floor surfaces, such as corridors, patient rooms, and toilets, were given a solid gray/beige color (stone/sand). Rooms where patients were not expected to be, such as offices, rinsing rooms, medication rooms, and the workstation, were given floors in different contrasting colors, mostly red. All floors were given solid colors since patterns and decorations can cause problems for the visually impaired, who might falsely perceive the floors as having level differences.

***The EBD intervention goals for color were to use colors to make it easier for users to find their way and gain information, thereby increasing safety and security.***

To indicate the boundaries of rooms, the floor was colored with a darker shade near the walls. The walls behind toilet seats and sinks were painted in contrasting colors to render them visible. Wall handrails made of wood, which contrasted with the color of the walls, were installed, as were doors of natural wood in emergency rooms.

Further EBD intervention goals for color included choosing durable and natural colors that would be perceived as recognizable over time. On the walls, natural and harmonious colors were chosen, and no trendy or fashionable colors were selected. Walls with medical equipment in treatment and patient rooms were painted white. If possible, natural materials, such as wood, were chosen. All colors and materials were selected based on the function of the rooms.

### Questionnaires

This study employed a self-report survey design in which questionnaires were distributed to patients upon arrival at the ED. The survey included the following: sample characteristics (Table 1), three validated questionnaires, the 12-Item Short-Form Health Survey (SF-12), and the Light and Color Questionnaire-Patient (LCQ-P) and Light and Color Questionnaire–Family-Member versions (LCQ-F; Lindahl et al., 2020).

The SF-12 is a health-related quality of life measure and the short version of the 36-item Short-Form Health Survey (SF-36). The SF-12 consists of 12 questions grouped into eight different health domains—physical functioning, role physical, bodily pain, general mental health, vitality, social function, role emotional, and general health. Participants completing the survey can obtain scores ranging between 0 and 100, with 0 reflecting the minimum health level and 100 the maximum health level (Al Omari et al., 2019; Ware et al., 1996). The SF-12 was used in this study to test the temporal stability of the participants’ health-related quality of life.

The LCQ-P and LCQ-F (Lindahl et al., 2020) are self-report questionnaires with a 6-point Likert-type scale (from 0 = *no, I disagree completely* to 5 = *yes, I agree completely*) designed to evaluate support from *light* and *color* in the physical environment of an ED. The LCQ-P and LCQ-F contains 11 items each: six statements for light (0–max 30) and five statements for color (0–max 25) with a focus on six dimensions derived from the Professional Environmental Assessment Protocol, namely, *maximizing awareness and orientation*, *maximizing safety and security*, *supporting functional abilities*, *providing privacy*, *opportunities for personal control* (opportunities for personal control excluded in the Color subscale), and *regulation and quality of stimulation*. A higher score indicates a higher level of support. The satisfactory content and internal validity (>90%) and high internal consistency (Cronbach’s alpha coefficient = .9) support the use of the LCQ-P and LCQ-F questionnaires for research and development purposes. A total of 600 questionnaire responses confirmed, through exploratory factor analysis, that light and color are distinct and independent dimensions that create perceptions of more or less supportiveness for respondents (Lindahl et al., 2020)

### Data Collection and Analysis

All patients who met the inclusion criteria after triage, as well as family members who visited the ED and met the selection criteria, had the opportunity to participate in the study until 100 surveys from patients and 100 surveys from family members were completed. This procedure was followed in both the before and after studies—200 surveys before and 200 surveys after refurbishment and remodeling. This yielded a total of 400 surveys: 100 + 100 before and 100 + 100 after. The before and after data collection procedures were conducted during February 2010 and 2012, respectively.

All patient and family member participants received written information about the study, its purpose, procedures, and how data would be handled. Participants were informed both orally and in writing that participation was voluntary and anonymous and they had the right to withdraw from the study at any time. The recent remodeling and refurbishment of the ED were not mentioned in the survey after the EBD intervention.

A completed survey was considered written consent to participate. The surveys were answered during the participants’ stay at the ED and returned in a sealed box before leaving the ED. The questionnaires were collected between 8 a.m. and 8 p.m. so that participants would be least affected by natural fatigue or sleepiness.

Descriptive statistics were used to present medians, means, standard deviations, and percentages. For comparisons between before and after refurbishment and remodeling, the Mann–Whitney U-test was used for ordinal variables. Statistical significance was set at *p* < .05. The data were analyzed both with and without imputed values. All analyses were performed using SPSS Version 21 (IBM, Corp., Armonk, NY, USA).

### Ethics

Ethical considerations as informed written consent, confidentiality, voluntariness, and risks/benefits were followed according to the Helsinki Declaration (World Medical Association, 2013). Ethical advice was given by Region Kronoberg Research Ethics Council (9/2009).

## Results

Before the EBD intervention at the ED in 2010, 100 patients and 100 family members responded to surveys. After the EBD interventionen at the ED in 2012, 100 patients and 100 family members responded to the same surveys. Thus, a total of 400 patients and family members participated in the study. The median age was 46–55 years for both patients and family members, more family members than patients were women, and the majority of patients and family members had visited the ED before (Table 1).

The median SF-12 score was the same in the before and after intervention groups for both patients and family members (Table 1). The only significant differences in background variables in the before and after intervention groups were seen in the distribution of visitors to different ED units: more patients visited the orthopedic department in the before group than in the after group (*p* = .04), and more family members visited the surgery department in the before group than in the after group (*p* = .05; Table 1).

**Table 1.** Characteristics of Participants Divided Into Patients and Family Members and the Type of ED Unit They Visited and Clinical Characteristics and SF-12 Median Scores.

	*Patient*			*Family Member*		
	*n* = 200			*n* = 200		
	Before *n* = 100	After *n* = 100	*p* Value	Before *n* = 100	After *n* = 100	*p* Value
*Age*-*group ^a^ median*	46–55	46–55	0.69	46–55	46–55	.61
*Age*						
* 18*–25	8	12		6	4	
26–35	12	8		9	12	
36–45	13	14		19	20	
46–55	16	14		19	21	
56–65	17	17		25	16	
66–75	17	19		16	20	
>*76*	16	16		6	6	
*Women/*m*en ^b^* (*n)* (*n*) %	56/39 59%/41%	50/49 51%/49%	.20	67/29 70%/30%	65/35 65%/35%	.54
*Earlier emergency department visits (n*)	82	77	.48	87	89	.83
*Emergency department unit* *n (%)*			.05			.04*
* Medicine*	36	46		38	45	
* Surgery*	24	17		28	14	
* Orthopedics*	26	13		22	15	
* Other*	8	14		4	8	
* Missing*	6	10		8	15	
*Median SF*-*12 score, 12-Item Short-Form Health Survey*	31 n = 87	31 n = 90	.59	32 n = 83	31 n = 75	.34

^a^ Age groups: 18–25 years, 26–35 years, 36*–*45 years,…to 76 years or more. Missing age: *n* = 1 before and *n* = 0 after.

^b^ Missing gender: *n* = 5 before and *n* = 1 after.

Significance: *p < .05, **p < .01, ***p < .001.

### Light: LCQ, Patient and Family–Member Versions

After the EBD interventionen of the ED, the perceived support from light, measured as the total score for the LCQ-P/F Light sections, was significantly higher for both patients and family members. The LCQ-P/F Light Section scores were significantly higher for three of six items for patients and four of six items for family members (Table 2).

***After the EBD interventionen of the ED, the perceived support from light, measured as the total score for the LCQ-P/F Light sections, was significantly higher for both patients and family members.***

The score for Item 4, *helps me feel private*, which assesses the provision of privacy, increased the most for both patients and family members after the intervention (Table 2).

**Table 2.** Scores for ***LIGHT*** of Light and Color Questionnaire-Patient (LCQ-P) and Family Member (LCQ-F) Before compared to after EBD intervention Emergency Department and Mann–Whitneys U-Test.

LCQ	Patient					Family Member				
*Light*	Before		After			Before		After		
	*n*	Median ^a^ Min–Max	*n*	Median ^a^ Min–Max	*p* Value	*N*	Median ^a^ Min–Max	*n*	Median ^a^ Min–Max	*p* Value
Light **Total score, Items 1***–***6**	88	**24** 0*–*30	99	**26** 0*–*30	.004**	93	**24** 0*–*3	95	**26** 0*–*30	.002**
Item		Median Mean (*SD*)		Median Mean (*SD*)			Median Mean (*SD*)		Median Mean (*SD*)	
Maximize **Awareness and Orientation**: Item 1: Helps me to find my way and orient myself	97	**4** **4.1** (1.0)	100	**5** **4.4** (0.7)	.032*	96	**4** **3.9** (1.2)	97	**4** **4.2** (0.9**)**	.09
Maximize **Safety and Security**: Item 2: Helps me to feel safe and secure	96	**4** **4.0** (1.1)	100	**5** **4.5** (0.7)	.004**	97	**4** **4.0** (1.0)	97	**5** **4.3** (1.0)	.046*
**Support functional abilities**: Item 3: Helps me so that I can move like I am used to	95	**4** **4.2 (**1.1)	100	**5** **4.4** (1.0)	.08	96	**4** **4.2** (0.9)	96	**5** **4.4** (1.0)	.06
Provision of **privacy**: Item 4: Helps me to feel private	91	**4** **3.3** (1.6)	99	**4** **3.9** (1.3)	.009**	98	**3** **3.0** (1.4)	96	**4** **3.7** (1.2)	.001**
**Opportunities for personal control**: Item 5: Helps me to be in control and have choices for my needs	97	**4** **3.8** (1.2)	100	**4** **4.1** (1.0)	.05	96	**4** **3.8** (1.2)	96	**4** **4.1** (1.0)	.033*
**Regulation and quality of stimulation**: Item 6: Gives me the opportunity to get the right light for my needs	96	**4** **3.9** (1.2)	100	**4** **4.2** (1.1)	.10	97	**4** **3.8** (1.2)	96	**4** **4.2** (0.9)	.014**

^a^ Min–max (0–5) for all Items 1–6.

Significance: *p < .05. **p < .01. ***p < .001.

### Color: LCQ, Patient and Family–Member Versions

After the EBD intervention of the ED, the perceived support from color, measured as the total score for the LCQ-P/F Color sections, was significantly higher for both patients and family members. The after scores for all five color items on the LCQ-P/F were significantly higher for both patients and family members (Table 3). The score for item 11 of the LCQ-P/F Color sections, which assesses regulation and quality of stimulation (*affects my visit positively*), increased the most for both patients and family members after the EBD intervention (Table 3). The internal response rate for each of the LCQ-P/F Light and Color sections was greater than 88%, and on the item level, it was 91%–100%.

***After the EBD intervention of the ED, the perceived support from color, measured as the total score for the LCQ-P/F Color sections, was significantly higher for both patients and family members. The after scores for all five color items on the LCQ-P/F were significantly higher for both patients and family members***

**Table 3.** Scores for COLOR of Light and Color Questionnaire-Patient (LCQ-P) and Family Member (LCQ-F) Before and After Refurbishing and Remodeling the Emergency Department and Mann–Whitneys U-Test.

LCQ	Patient					Family Member				
*Color*	Before		After			Before		After		
	*n*	Median ^a^ Min–Max	*n*	Median ^a^ Min–Max	*p* Value	*n*	Median ^a^ Min–Max	*n*	Median ^a^ Min–Max	*p* Value
Color **Total score, Items 7***–***11**	91	**18** 0*–*25	**98**	**20** 0*–*25	<.001***	98	**16** 0*–*25	95	**20** 0*–*25	<.001***
Item		**Median Mean (*SD*)**		**Median** **Mean (*SD*)**		n	**Median** **Mean (*SD*)**	n	**Median** **Mean (*SD*)**	
**Awareness and Orientation**: Item 7: Helps me to find my way and orient myself	96	**4** **3.5** (1.5)	99	**4** **4.0** (1.0)	.009**	100	**4** **3.3** (1.4)	96	**4** **3.8** (1.1)	.002**
**Safety and Security**: Item 8: Helps me to feel safe and secure	94	**4** **3.4** (1.4)	99	**4** **4.1** (1.0)	.001**	98	**3** **3.3** (1.4)	96	**4** **3.8** (1.2)	.005**
**Support functional abilities**: Item 9: Helps me so that I can move like I am used to	96	**4** **3.5** (1.4)	99	**4** **4.0** (1.0)	.006**	98	**4** **3.4** (1.4)	96	**4** **4.0** (1.1)	.002**
Provision of **privac**y: Item 10: Helps me to feel private	93	**3** **2.9** (1.7)	98	**4** **3.7** (1.3)	.002**	98	**3** **2.9** (1.5)	95	**4** **3.5** (1.4)	.003**
**Regulation and quality of stimulation**: Item 11: Affects my visit positively	95	**3** **3.1** (1.5)	99	**4** **4.0** (1.2)	<.001***	100	**4** **3.3** (1.4)	96	**4** **3.8** (1.1)	.002**

^a^ Min–Max (0*–*5) for all Items 1*–*6.

Significance: *p < .05. **p < .01. ***p <.001.

## Discussion

This study of an EBD intervention focusing on altering light and color by refurbishing and remodeling an ED in south Sweden revealed a significantly higher score for self-reported support from light and color for patients and family members after the EBD intervention compared to before. We used the LCQ-P/F versions to assess whether and the degree to which light and color were perceived as supportive in the physical care environment (Lindahl et al., 2020).

The Light sections on the LCQ-P and LCQ-F yielded higher overall scores than did the Color section of the LCQ-P and LCQ-F both before and after the EBD intervention; however, the scores for Color section of the LCQ-P and LCQ-F increased the most. The scores for the dimensions of *privacy* and *safety and security* increased significantly for the Light section of both the LCQ-P and LCQ-F. The score for the dimension of *awareness* and *orientation* increased significantly for the LCQ-P Light section, and the scores for the dimensions of *opportunities for personal control* and *regulation and quality of stimulation* increased significantly for the Light section of the LCQ-F. The scores for all five dimensions of the Color section of the LCQ-P and LCQ-F—*awareness and orientation, safety and support, functional abilities, privacy, and regulation and quality of stimulation*—increased significantly after the EBD intervention.

It seems that the amendments of light and color to support patients and family members in the physical care environment, which were the goal of the EBD intervention evaluated in this study, were not isolated from each other; rather, they were both components of the support since both the Light and Color sections of the LCQ-P and LCQ-F yielded increased scores for perceived support after the EBD intervention. This is consistent with the necessity of light and color working together to allow us to perceive and understand the world (Klarén, 2017; Mahnke, 1996). Both patients and family members perceived a significant increase in support by *light*, which helped them to feel *safe* and *secure*, as well as provide them with *privacy*. This aligns with evidence that indicates that lighting is important in the physical environment (Caspari et al., 2011; Iyendo et al., 2016), and its contribution to feeling safe and secure has been described previously (Anåker et al., 2017).

The EBD intervention aimed at amending lighting at the studied ED included more and new windows equipped with adjustable blinds in walls and doors to provide access to natural daylight and brightness from the space outside the rooms. More windows create a dynamic interior space that supports the diurnal cycle, as well as provides a view of the outside (Caspari et al., 2011; Engwall et al., 2015; Ulrich et al., 2008), and, according to previous studies, promotes independence, safety, and security (Anåker et al., 2017). Access to nature via outside views stimulates positive emotions and well-being and decreases negative emotions (Iyendo et al., 2016; Kellert & Calabrese, 2015). The lighting EBD intervention also affected the dimensions of *safety and security* and *providing privacy* by installing different light sources, such as spotlights, mood lights, and general lighting in patient rooms, waiting rooms, and corridors, further giving patients and family members the choice, by an on/off switch, of enjoying one or several light sources at the same time. All patient rooms and many other rooms had on/off switches and offered the selection of different light values by dimmer switch after the intervention. Before the intervention, patient rooms had only fluorescent lights directly over the bed. Lying in a bed with bright lighting has been assessed as both unpleasant and not promoting privacy (Annemans et al., 2018). Lighting design that supports patients and family members allows them to decide on their individual lighting needs, which is consistent with guidelines for intensive care unit design (Thompson et al., 2012). Shadows may provide emotional security, whereas too much light can aggravate negative emotions. Moreover, a physical care environment that is always bright is unpleasant for patients in need of rest. The right lighting can change the impression of the physical environment, rendering it more attractive, welcoming, restful, or stimulating (Iyendo et al., 2016; Caspari et al., 2007).

Significantly higher support from light was perceived as affecting *awareness and orientation* after the EBD intervention compared to before, according to LCQ-P Light scores; however, the LCQ-F Light scores did not improve significantly. This difference may be explained by patients often being in bed, whereas family members are often sitting and able to walk around in the ED. A view through hospital room windows helps coding, orientation, and navigation and also promotes independence within hospital premises (Anåker et al., 2017; Iyendo et al., 2016).

Patients and family members frequently encounter EDs that are overcrowded and have long wait times; such ED experiences create feelings of dependency, fear, anxiety, vulnerability, and lack of control (Elmqvist & Frank, 2015; Gordon et al., 2010; Sonis et al., 2018). Furthermore, perceived self-image can change when patients and family members enter the ED, transitioning from being previously independent and healthy to becoming ill or injured in a patient’s role associated with feelings of ignorance, uncertainty, and loss of control (Dahlen et al., 2012). Thus, it would be supportive if patients and family members at least had control of the type of lighting they need, whether they choose bright lighting for full visibility of themselves and a detailed view of their surroundings or a less well-lit environment to be less visible and enjoy the privacy connected with darkness.

The scores for the LCQ-F Light section improved significantly for the dimensions of *regulation and quality of stimulation* and *opportunities for personal control*; however, the corresponding scores for the LCQ-P Light section did not improve after the EBD intervention. A plausible explanation for this discrepancy is that light switches and dimmers were placed near doors and blind controls near windows, and neither of them could be controlled from the patient’s bed. Thus, when planning a physical care environment, control equipment can be advantageously placed easily accessible to visitors and, especially, patients.

The use of colors in the design of physical care environments should be based on current knowledge, with an awareness of the effects, influences, and possibilities connected with color (Caspari et al., 2011). Dalke (2006) emphasized that color attracts more attention than form, especially for children.

The EBD intervention involving color at the studied ED provided color contrasts on walls and floors, solid natural colors without patterns on all floors and walls, and included wooden materials, which all are seen as pleasant and independent of modern trends or fashions, whish after some years could been regarded as old and out of date. Studies have shown that patients judge care based on how they experience the care environment. A hospital environment that is old, worn or in poor condition/unclean, the care judged by patient to be inadequate and that the staff does not care. (Edvardsson et al., 2005).

Color and color contrasts can be supportive when used to direct attention and facilitate the interpretation of the environment in order to prevent accidents and establish spatial clues for awareness and control (Iyendo et al., 2016). Furthermore, color may evoke emotional and physiological reactions that can aggravate or relieve stress, elicit emotional states (e.g., calmness), play a role in the care process, contribute to a sense of well-being, and support safety and independence (Davis & Weisbeck, 2016; Mahnke, 1996; Wijk, 2006. However, the use of color in EBD as implemented in the studied intervention, namely, remodeling and refurbishing an ED to improve support for both patients and family members, is new knowledge that could be useful for assessing perceptions of support from the physical environment in other healthcare settings as well.

### Strengths and Limitations

Few instruments exist for assessing the quality of the physical environment in healthcare which have been subjected to satisfactory psychometric procedures (Elf et al., 2017), and no validated instruments exist for the assessment of support light and color from the user’s perspective. However, in this study, the validated instrument LCQ is used (Lindahl et al., 2020).

Although the data for this study were collected in 2012, no studies have been found to date that were investigating perceived support from light and color in general nor from the perspective of patients and family members in particular. Hence, the EBD intervention is novel and the results, interpretations, and implications are new and adding to the literature. Our study intended to link the perceived support from light and color before and after the EBD intervention at the ED, not to the remodeling and rebuilding itself. Furthermore, the focus was the perceived support from the user’s perspective.

A quasi-experimental design was chosen for this study (Portney, 2020), as patients and family members were not the same before and after the EBD intervention. However, a limitation that could not have been amended, since patients visit EDs only when they are acutely ill or injured. Therefore, it was not possible to randomize participants into intervention and control groups in this type of before-and-after study.

Age, gender distribution, and self-reported health-related quality of life obtained using the SF-12 did not differ in the before and after conditions among respondents; this indicates, but does not prove, that the improved LCQ scores might be attributable to the EBD intervention even though it was not the same 100 consecutive patients and 100 family members who responded to the preintervention and postintervention LCQ questionnaires. According to the study design, data collection was completed when 100 patients and 100 family members had responded in the before and after studies in order to make the two groups comparable in size. The study, therefore, had no dropouts. Selection bias can thus be expected; however, we cannot find logical reasons for the selection of participants to be different in the before versus after data collection. The remodeling and refurbishment of the ED in the before condition was not mentioned in the information provided to participants in the after study.

Significantly, more patients and family member participants were recruited from the orthopedic and surgery ED units in February 1 year before the intervention than in February 1 year after the intervention. This could be an explained by in February in the before study than February in the after study since this type of weather is associated orthopedic injures (Vergouwen et al., 2021). However, the same ED rooms were used for all patients, and the staff was similar in both years with respect to the number of nurses and physicians in service.

The positive changes in perception of light and color at the ED found in our study may be attributed to the sheer newness of the environment reached by the intervention and not to changes in light and color as such. This “newness” factor may resemble the Hawthorne effect (Merrett, 2006). This potential bias was tested by a sensitivity analysis comparing changes in LCQ-score based on whether the participants had visited the ED before or not. No obvious differences between LCQ-score changes related to prior ED visits were detected.

## Conclusion

This study showed improved perceived support from light and color in the physical environment for patients and family members following an EBD intervention at an ED. The assessment of the perceived support was conducted using a validated instrument: the LCQ.

## Implications for Practice

The main implications for practice are as follows:

- Since the self-reported questionnaire LCQ assessing how light and color are perceived showed good validity, it could be used to assess and improve the design of future healthcare environments.
- The results from this study of an EBD intervention could be used for informing policies and guidelines on design of healthcare environments.
- Knowledge from this EBD intervention could also be useful for architects, administrators, and interdisciplinary research.

**Authors’ Note:** Region Kronoberg ethics committee approved this study (9/2009).

## Supplemental Material

Supplemental Material, sj-pdf-1-her-10.1177_19375867221150215 - The Perceived Support From Light and Color Before and After an Evidence-Based Design Intervention in an Emergency Department Environment: A Quasi-Experimental Study Click here for additional data file. Supplemental Material, sj-pdf-1-her-10.1177_19375867221150215 for The Perceived Support From Light and Color Before and After an Evidence-Based Design Intervention in an Emergency Department Environment: A Quasi-Experimental Study by Jeanette Lindahl, Hans Thulesius, Helle Wijk, David Edvardsson and Carina Elmqvist in HERD: Health Environments Research & Design Journal
