# Supplementary material for: The Perceived Support From Light and Color Before and After an Evidence-Based Design Intervention in an Emergency Department Environment: A Quasi-Experimental Study
Source: HERD. 2023 Mar 2;16(2):109–24. doi: 10.1177/19375867221150215 (PMC10133835; doi:10.1177/19375867221150215)
Supplement: Supplemental Material, sj-pdf-1-her-10.1177_19375867221150215 - The Perceived Support From Light and Color Before and After an Evidence-Based Design Intervention in an Emergency Department Environment: A Quasi-Experimental Study [file sj-pdf-1-her-10.1177_19375867221150215.pdf]

Jeanette Lindahl

Forskningsetiska rådet

Beslutsdatum 2009-11-18 **Beslutsnummer 9/2009**

Beslutsärende pkt 2

Ansökan om forskningsetisk rådgivning avseende "*Skapande av en god vårdmiljö på akutkliniken CLV*" har inkommit från Jeanette Lindahl 2009-11-05.
Studien har rubricerats som ett utvecklingsprojekt.

Akutkliniken CLV står inför en omfattande ombyggnation och möjlighet finns att tillämpa och utveckla den forskning om god vårdmiljö som genomförts under de senaste åren.

Syftet med studien är att beskriva och förstå sinnesintryckens, framförallt färgernas och ljusets betydelse, för att främja människors hälsoprocesser och skapa stödjande vårdmiljöer inom akutsjukvården.

Forskningsetiska rådet tycker det är oklart om studien ska bedömas som ett utvecklingsprojekt eller ett forskningsprojekt. I ansökan uppger sökande att studien är utvecklingsprojekt men i informationsbreven beskrivs studien som ett forskningsprojekt.

Forskningsetiska rådet bedömer att projektet ur forskningsetisk synpunkt inte behöver genomgå prövning av forskningsetikkommitté eftersom uppgifterna inte kan kopplas till enskild person enligt Personuppgiftslagen (PUL).

Oavsett om studien är att betrakta som forsknings- eller utvecklingsprojekt lämnar forskningsetiska rådet synpunkter som vi hoppas ska kunna bidra till att förbättra studiens kvalitet.

Urval
Urvalet bör beskrivas tydligare. Hur väljer man ut patienter och närstående? Konsekutivt, strategiskt eller slumpmässigt?
När under besöket kommer patient/närstående att fylla i enkäten?

Finns det en plan för hur man ska hantera bortfall? Bortfallsprotokoll bör utarbetas, särskilt viktigt om studien ska vara del i forskningsprojekt.

Vårdpersonal som själva ingått i studien och som lämnar ut enkät till patient eller närstående kan sannolikt påverka information och motivation. Detta innebär att enkätsvaren måste bedömas med denna skevhet i åtanke. Kanske skulle vård-personalens hantering av information till deltagande patienter och närstående standardiseras genom att en checklista / ett PM utformas?

Komplettering bör göras med demografiska data som ålder, kön, besöksorsak, tid för besöket (för att få uppfattning om dygnsvariation). Genom att samla in demografiska data beskrivs deltagarna på ett sätt som underlättar tolkning av resultaten.
Dessutom bör man då kunna avstå från att använda SF-12, för att göra enkäten lättare att besvara.

Instrument
Om studien genomförs som ett utvecklingsprojekt föreslår vi att de viktigaste påståendena ur vårdklimatinstrumentet väljs ut så det högst upptar en A-4-sida.
Detta eftersom vi uppfattar att vårdklimatinstrumentet är komplicerat att besvara.
Därmed riskeras en låg svarsfrekvens i studien, dvs ett högt internt bortfall – att enkäten inte besvaras i tillräcklig omfattning.

Om studien genomförs som ett forskningsprojekt bör inga ändringar göras på vård-klimatinstrumentet eftersom forskning som använder validerade instrument är lättare att publicera och få godkänd som doktorandarbete.

Rådet har inga etiska invändningar angående användandet av SF-12 men vad är syftet?
Ska även vårdare fylla i SF-12?
Bättre än att använda SF-12 vore att registrera demografiska data enligt ovan för att beskriva deltagarna i studien. Även detta för optimering av intern svarsfrekvens genom att göra enkäten lättare att besvara.

Dokumenten med frågor om Ljus och Färg bör omarbetas.
Komplettera med en inledande instruktion. Använd samma begrepp genomgående, nu används ljus-ljussättning-belysning och färg-färgsättning. Begreppen "ljus" respektive "färg" bör användas istället för ljussättning eller färgsättning.
Mer vardagligt språkbruk gör enkäten lättare att besvara.
Komplettera formulären så att den som svarar ges möjlighet att skriva egna kommentarer efter varje fråga om ljus och färg. På så vis uppmuntrar man deltagarna att med egna ord berätta vad man egentligen tycker om vårdmiljön.
Man har ofta mer nytta av fria kommentarer än av förformulerade frågor om syftet är att förbättra en verksamhet.
Fria kommentarer kan, om de ges tillräckligt utrymme i enkäten, ge tillräckligt med data för att analyseras vetenskapligt.
Ofta svarar 20-30% med egna kommentarer vilket i denna studie skulle kunna innebära öppna svar från minst 50 personer.

Information
Om studien genomförs som ett utvecklingsprojekt ska informationsbreven ändras, i texten står det forskningsprojekt.

Samtliga informationsbrev bör förbättras både när det gäller layout och språkbruk.

Enligt uppdrag

Yvonne Thörnemyr
Sekreterare
Forskningsetiska rådet
